# Activity and phylogenetic diversity of sulfate-reducing microorganisms in low-temperature subsurface fluids within the upper oceanic crust

**DOI:** 10.3389/fmicb.2014.00748

**Published:** 2015-01-14

**Authors:** Alberto Robador, Sean P. Jungbluth, Douglas E. LaRowe, Robert M. Bowers, Michael S. Rappé, Jan P. Amend, James P. Cowen

**Affiliations:** ^1^NASA Astrobiology Institute, University of HawaiiHonolulu, HI, USA; ^2^Hawaii Institute of Marine Biology, School of Ocean and Earth Science and Technology, University of HawaiiKaneohe, HI, USA; ^3^Department of Oceanography, School of Ocean and Earth Science and Technology, University of HawaiiHonolulu, HI, USA; ^4^Department of Earth Sciences, University of Southern CaliforniaLos Angeles, CA, USA; ^5^Department of Biological Sciences, University of Southern CaliforniaLos Angeles, CA, USA

**Keywords:** subseafloor life, metabolic activity, functional diversity, sulfate reduction, basaltic ocean fluids

## Abstract

The basaltic ocean crust is the largest aquifer system on Earth, yet the rates of biological activity in this environment are unknown. Low-temperature (<100°C) fluid samples were investigated from two borehole observatories in the Juan de Fuca Ridge (JFR) flank, representing a range of upper oceanic basement thermal and geochemical properties. Microbial sulfate reduction rates (SRR) were measured in laboratory incubations with ^35^S-sulfate over a range of temperatures and the identity of the corresponding sulfate-reducing microorganisms (SRM) was studied by analyzing the sequence diversity of the functional marker dissimilatory (bi)sulfite reductase (*dsrAB*) gene. We found that microbial sulfate reduction was limited by the decreasing availability of organic electron donors in higher temperature, more altered fluids. Thermodynamic calculations indicate energetic constraints for metabolism, which together with relatively higher cell-specific SRR reveal increased maintenance requirements, consistent with novel species-level *dsrAB* phylotypes of thermophilic SRM. Our estimates suggest that microbially-mediated sulfate reduction may account for the removal of organic matter in fluids within the upper oceanic crust and underscore the potential quantitative impact of microbial processes in deep subsurface marine crustal fluids on marine and global biogeochemical carbon cycling.

## Introduction

Exploratory studies have revealed that low-temperature fluids flowing within the uppermost basaltic ocean crust harbor free-living microorganisms that are only distantly related to any cultivated strains or communities attached to basaltic surfaces (Jungbluth et al., 2013). In fluids from the basaltic ocean crust, metabolisms of free-living populations related to oxygen, iron, sulfur, hydrogen, and methane cycling have been inferred from fluid alteration chemistry (Wheat et al., 2010; McCarthy et al., 2011; Orcutt et al., 2013; Lin et al., 2014) and sequence diversity in environmental DNA (Cowen et al., 2003; Huber et al., 2006; Jungbluth et al., 2013, 2014). However, owing to the wide range of conditions in the ridge flanks and the severely restricted access to crustal aquifers, rates of catabolic activity have only been predicted (Johnson et al., 2006; Orcutt et al., 2011, 2013).

Microbial populations and their rates of catabolic activity in basaltic fluids are constrained by the substrate diversity and availability, e.g., electron donors and acceptors, which in turn depend on the nature of the fluid flow regimes (Cowen, 2004). Simple examples of these are found on the eastern flank of the Juan de Fuca Ridge (JFR). For instance, at transition sites from sediment free to sediment covered basaltic crust, a rapid lateral fluid flow or mixing has been inferred to occur from ventilated to isolated hydrothermal conditions (Davis and Becker, 2002). Furthermore, as sediment thickness approaches ~160 m, laterally flowing fluids remain largely isolated, experiencing increasing temperatures as they move away from the spreading ridge (Spinelli and Fisher, 2004). These fluid flow regimes limit the generation and circulation of key nutrients (e.g., nitrogen and phosphorus), dissolved inorganic and organic carbon, and electron acceptors and donors (Elderfield et al., 1999; Lang et al., 2006; Walker et al., 2008), resulting in geochemical alterations that have uncertain consequences for the distribution of microbial life.

Here we study microbial sulfate reduction and examine the thermal and energetic factors that limit the distribution and activity of sulfate-reducing microorganisms (SRM) in subsurface crustal fluids within the flow paths of the hydrogeologically active upper oceanic crust. Microbially-mediated sulfate reduction is a globally important redox process in anoxic marine sediments (Canfield et al., 2005) but unexplored in the oceanic lithosphere. We used recently developed seafloor sampling systems (Lin et al., 2012) to collect large volumes of high integrity crustal fluids from Circulation Obviation Retrofit Kit (CORK) observatories (Fisher et al., 2005) for experimental procedures. Our sulfate-reduction rate measurements and sulfate-reducing microbial community analysis are indicative of the metabolic potential and broad dispersal of organotrophic sulfate-reducing microbial groups along fluid flow paths within deeply buried basaltic crust with significant implications for the marine carbon cycling.

## Materials and methods

### Sampling of basaltic fluids

CORK observatories seal the top of the casing in an Ocean Drilling Program (ODP/IODP) reentry cone installation to prevent circulation between the open hole and ocean bottom water allowing the access to crustal fluids (Fisher et al., 2005). Fluids were sampled as previously described using a mobile pump system (Lin et al., 2012) to distribute fluids from the CORK fluid delivery lines into custom made large volume (up to 60 L) gas sampling bags. Sampling bags were made of Tedlar™ polyvinyl fluoride (PVF) film with heat-sealed, leak-proof, double seams (MiDan Co., Chino, CA, USA). Fluids were collected into sample bags once the fluid temperature exiting the fluid delivery line rose to a stable temperature, indicating that the line had been sufficiently flushed. Typically, flushing of fluid delivery lines was allowed long enough to replace at least three times the volume of borehole fluids residing inside. Temperature and flow sensors installed in the sampling system allowed monitoring of the sampling process. Fluid samples were collected in the summer of 2010 over four consecutive dives using a deep-sea submersible, the remotely operated vehicle (ROV) *Jason II*. In the present study, one sample bag collected at CORK 1025C (JFR #10), and three sample bags collected at CORK U1301A (JFR #4, #5, and #15), were used. The sampling bags were mounted on an independent elevator that was returned to the ship within an hour after fluid collection. Extremely low O_2_ permeability (3.2 cm^3^ 100 in^−2^ 24 h^−1^ atm^−1^ mil^−1^, at 25°C) and quick sample turnover of sample bags insured minimum oxygen diffusion into the bags. Shipboard chemical analysis showed no significant oxidation of fluids (Lin et al., 2012). Additionally, deep bottom seawater was collected for background controls in the vicinity of CORK 1025C using the bag sampling system, and in the vicinity of U1301A using a conductivity, temperature, and depth (CTD) Niskin-rosette.

### Geochemical characterization of fluids

Fluids from CORKs 1025C and U1301A were analyzed for all of the major and minor chemical constituents in seawater. Analytical data used in this study are compiled in Table 1. Additional original data, including analytical methods, are published elsewhere (Lin et al., 2012; Jungbluth et al., 2014).

**Table 1:** **Chemical composition of basement fluids collected from boreholes 1025C and U1301A**.

	**1025C**	**U1301A**
Seafloor depth (m)	2606.2	2667.3
Pressure (kPa)	26,555	27,071
Temperature (°C)	39	63
pH	7.9	7.4
**DISSOLVED CONSTITUENTS**		
Mg^2+^ (mM)	29.7	1.9
Ca^2+^ (mM)	30.4	54
Alkalinity (meq/L)	0.88	0.46
NO^−^_3_ (μM)	6.4	0.3
NO^−^_2_ (μM)	<0.05	<0.05
NH^+^_4_ (μM)	43	103
SO^2−^_4_ (mM)	26.2	17.2
Cl^−^ (mM)	539	557
Na^+^ (mM)	469	471
K^+^ (mM)	9.4	6.3
Si (μM)	590	1166
PO^3−^_4_(μM)	0.05	0.06
Fe^3+^ (μM)	1.3	0
Fe^2+^ (μM)	1.2	0.9
Mn^2+^ (μM)	1	4.1
Mn^4+^ (μM)	1.2	0.6
DOC (μM)	22	13
H_2_S (μM)	0	0.17

### Measurement of sulfate reduction rates (SRR)

Upon recovery on deck, 50 ml subsamples were collected from sample bags JFR #10 (1025C) and JFR #4 (U1301A) and were immediately transferred under N_2_ into serum bottles and incubated in the dark with 100 kBq/ml of ^35^S-labeled sulfate using a thermal gradient block. SRR were determined in duplicates on time-course experiments every 24 h. Activity in 1025C and U1301A fluids was measured above minimum detection limit after a maximum incubation period of 3 and 5 days, respectively. Incubation times were reduced in order to minimize potential cell growth and re-oxidation of the radiolabeled sulfide. Incubation experiments were carried out with unamended fluids as well as with fluids amended with inorganic (H_2_) and organic electron donors (a mixture of short-chain volatile fatty acids; acetate, butyrate, propionate, and lactate to a final concentration of 1 mM). Following the incubation, the samples were fixed with 25 ml of a 5% zinc acetate solution and stored frozen at −20°C until further processing. Reduced sulfur was extracted back in the laboratory from the sample by a single-step HCl and chromium distillation method (Kallmeyer et al., 2004; Røy et al., 2014). The detection limit of SRR was estimated as previously described (Kallmeyer et al., 2004; Røy et al., 2014) taking into consideration the total sample background that is attributable to all the background sources (i.e., counter, distillation, and tracer backgrounds). Additionally, the count rate inherent to a hypothetical contamination of sampled fluids with bottom seawater was added to the total sample background. The bottom seawater background was determined by distilling bottom seawater controls following the same experimental procedures as for basaltic fluids.

### Thermodynamic modeling

The potential energy yields for sulfate reduction with organic matter in 1025C and U1301A CORK fluids were evaluated by calculating the Gibbs energy of reaction (Δ*G_r_*) at the experimental temperature conditions according to:

$$\Delta G_{r}=\Delta G_{r}^{{}^{\circ}}+RT\text{ ln}Q_{r}$$

where Δ*G_r_*° stands for the standard state Gibbs energy of reaction at the temperature and pressure of interest, *Q_r_* represents the reaction quotient, *R* refers to the gas constant, and *T* corresponds to the temperature in Kelvin. Activities of the chemical species required to calculate values of *Q_r_* were computed using the concentrations of the relevant species and their individual ion activity coefficients at the *in situ* temperatures (Lin et al., 2012; Jungbluth et al., 2014). Acetate was used as a proxy for organic matter in the reaction with sulfate. Values of Δ*G_r_*° were calculated using the revised HKF equations of state (Tanger and Helgeson, 1988; Shock et al., 1992), the SUPCRT92 software package (Johnson et al., 1992) and thermodynamic data from Shock and Helgeson (1990), Sverjensky et al. (1997), Helgeson et al. (1998), Dick et al. (2006), LaRowe and Helgeson (2006). Differences in net metabolic energy yield for sulfate reduction between both sampled sites are best exemplified when values are normalized per kg of crustal fluid. Values of Δ*G_r_* were normalized per kg of basaltic fluid by multiplying these values by the concentration of the limiting reactant, which was that with the lowest concentration after accounting for stoichiometry. This is meant to demonstrate the metabolic energy yield of the reaction of organic matter with sulfate in both 1025C and U1301A sampled fluids. Exergonic processes are denoted with positive values of Δ*G_r_*.

### Sampling for molecular microbiology and DNA extraction

Upon recovery on deck, 2.0, 5.3, 14.9, and 29.1 L of fluids collected from large volume samples JFR sample #10 (1025C), JFR sample #4, 5, and 15 (U1301A), respectively, were filtered through 0.22-μm-pore-sized Sterivex-GP filter cartridges (Millipore, Billerica, MA, USA), and subsequently stored in 2.0 ml of DNA lysis buffer [20 mM Tris-HCl, 2 mM EDTA, 1.2% Triton X-100, 2% lysozyme (w/v), pH 8] at −80°C until further processing. Background bottom seawater (4.0–4.3 L and 4.9–5.0 L collected in the vicinity of 1025C and U1301A, respectively) was processed as described for basaltic fluid samples.

Membrane filters were thawed to room temperature prior to nucleic acid extraction. Environmental DNA from filter membranes was extracted using the PowerSoil DNA isolation kit (MoBio Laboratories, Carlsbad, CA, USA) following the manufacturer's protocol. JFR sample #15 was extracted using a modified lysis and purification method similar to Frias-Lopez et al. (2008). A 40 μl solution of 50 mg ml^−1^ lysozyme (Sigma-Aldrich, St. Louis, MO, USA) in DNA lysis buffer was added to the Sterivex filter and incubated at 37°C rotating for 45 min. Proteinase K (Qiagen, Valencia, CA, USA) was added to a final concentration of >0.55 μAU and 20% SDS (Fisher Scientific, Waltham, MA, USA) was added to a final concentration of 1% and incubated at 55°C rotating for 2 h. Lysate was transferred to a 30 ml Oak Ridge tube using a sterile syringe. An additional 1 ml of lysis buffer was added to the filter for washing at 55°C for 15 min and pooled with above lysate. Phenol:chloroform:isoamyl alcohol (25:24:1; pH 8.0) totaling 3 ml was added and the mixture vortexed for 30 s and spun for 5 min at 2500 × g. The aqueous phase was transferred to a new Oak Ridge tube and 3 ml of chloroform:isoamyl alcohol (24:1) was added and the mixture vortexed for 30 s and spun for 5 min at 2500 × g. The aqueous phase was concentrated by spin dialysis using an Amicon Ultracel-30K filter (Millipore) and spinning at 1000 × g for 20 min. Flow-through was decanted and the Amicon filter was spun at 1000 × g for 20 min. A 1 ml volume of TE buffer [10 mM Tris-HCl (pH 8.0), 1 mM EDTA (pH 8.0)] was added over the Amicon filter membrane and spun at 1000 × g for 10 min; ~700 μl remained on the column and was transferred to a new tube. The filter column was washed twice with 700 μl of TE buffer and pooled with above DNA concentrate. Nucleic acids were concentrated using a vacuum centrifuge, resuspended in 50 μl of PCR-grade water and pooled together. Quantification of genomic DNA was performed using a Quant-iT™ dsDNA Assay High Sensitivity Kit (Q33120, Life Technologies, Carlsbad, CA, USA).

### Dissimilatory (Bi)sulfite reductase (dsrAB) gene cloning and sequencing

Whole-genome amplification (WGA) of environmental DNA extracts was performed prior to PCR using the Illustra GenomiPhi V2 DNA Amplification Kit (GE Healthcare Bio-Sciences, Piscataway, NJ, USA) following the manufacturer's instructions. In order to effectively eliminate the amplification of exogenous DNA, multiple displacement amplification (MDA) reagents, including the Phi29 polymerase and random hexamer primers, were UV-irradiated immediately before use following a previously published protocol (Woyke et al., 2011). Briefly, sample buffer, reaction buffer, and enzyme mix were UV-irradiated (254 nm) on ice for 30 min using a CL-1000 UV-crosslinker (Stratagene, La Jolla, CA, USA). WGA reactions were conducted for 1.5 h and included negative controls.

Amplification of full-length *dsrAB* was performed using WGA nucleic acids from sample JFR #15 using previously developed (Steger et al., 2011) degenerate primers DSR1Fmix (equimolar mixture of 10 μM each DSR1F variant) and DSR4Rmix (equimolar mixture of 10 μM each DSR4R variant) (Tables S1, S2). PCR reactions (25 μl) were prepared with PrimeSTAR Max premix (Takara Bio Inc., Otsu, Shiga, Japan). Hot-start PCR (i.e., addition of the template DNA to pre-heated reaction components) was used to minimize nonspecific amplification products (D'Aquila et al., 1991). The first round of amplification employed touchdown PCR using an initial denaturation step at 95°C for 3 min, followed by 10 cycles at 95°C denaturation for 30 s, 58°C to 48°C annealing for 30 s (the temperature was reduced by 1°C after each cycle), and 72°C elongation for 1 min 10 s. An additional 35 cycles at a constant annealing temperature of 48°C were performed prior to the final extension step at 72°C for 3 min. One microliter of PCR product was used as template for a second round of PCR applying an initial denaturation step at 95°C for 3 min, followed by 45 cycles of 95°C denaturation for 30 s, 48°C annealing for 30 s, and 72°C extension for 1 min 10 s. The cycling was completed by a final 72°C extension step for 3 min. Amplification of full-length *dsrAB* was successful only for the sample collected from the largest volume filtration (JFR #15); repeated attempts with samples JFR #4, 5, 10 and seawater controls were unsuccessful using a variety of thermocycling conditions (Table S2).

Amplification of *dsrB* fragments was performed using whole-genome amplified nucleic acids from all basaltic fluid samples (JFR #10, #4, #5, and #15) and bottom seawater background controls following a nested approach and using a primer set previously proved successful for basaltic rock samples (Lever et al., 2013), modified to include the addition of forward primer dsrB F1i (Table S1), which was generated using information from the successful DNA sequencing of full-length *dsrAB*. Two successive hot-start PCRs were performed as described above, with the exception that primers DSRB F1mix (equimolar mixture of 10 μM each DSRB F variant) and DSRB 4RSImix (equimolar mixture of 10 μM each DSRB 4RSI variant) (Table S1) were used in the second PCR reaction. Negative control PCR reactions yielded no visible product on an agarose gel.

Amplified fragments of anticipated length were excised from a 1% agarose gel and subsequently purified using the QIAquick gel extraction kit (Qiagen). Replicate *dsrAB* and *dsrB* PCR products were pooled together and cloned using the BigEasy v2.0 Long PCR Cloning Kit (Lucigen, Middleton, WI, USA). Clones were sequenced bidirectionally (*dsrAB*) or unidirectionally (*dsrB*) on an ABI 3730XL DNA Analyzer (Applied Biosystems, Carlsbad, CA, USA). Internal sequencing of *dsrAB* was performed using previously developed internal primers dsr619F (5′-GYCCGGCVTTCCCSTACAA-3′) and dsr1905BR (5′-ATGTGCGGCGCSGTDCAY-3′) (Giloteaux et al., 2010).

### dsrAB and dsrB gene analysis

DNA sequences were trimmed of vector sequence and manually curated using Sequencher version 5.1 software (GeneCodes, Ann Arbor, MI, USA). Full length *dsrAB* sequences were assembled using Sequencher default parameters. Clone sequences were aligned using the ARB software package (Ludwig et al., 2004) “integrated aligners” tool with a previously published database of aligned *dsrAB* sequences (Loy et al., 2009). Additional sequences that were most similar to clone sequences obtained in this study, as revealed by BLAST search against the non-redundant nucleotide database (Altschul et al., 1990), were aligned as described above and included in relevant phylogenetic analyses. A statistical selection of best-fit models of nucleotide substitution was performed on the DNA sequence alignment using jModelTest (Darriba et al., 2012) version 2.1.1. Phylogenetic analyses were performed with the RAxML maximum likelihood method using the GTR model of nucleotide substitution under the gamma- and invariable-models of rate heterogeneity (Stamatakis, 2006). Trees with the highest log likelihood score were selected from performing 100 iterations of the RAxML method. Phylogenies for the guide tree (**Figure 5**) and *Archaea* tree (Figure S1) were constructed initially using near full-length *dsrB*; sequence fragments of short length (<300 nt) were added to the maximum likelihood-derived phylogenies using the parsimony insertion tool in ARB (Ludwig et al., 2004). Phylogenies for **Figures 6, 7** were generated using sequence alignments of unambiguously aligned nucleotide positions (268 nucleotides) overlapping with the *dsrB* fragments generated in this study. Bootstrap analyses were determined by RAxML using the rapid bootstrap analysis algorithm (1000 bootstraps) implemented within ARB (Stamatakis et al., 2008). All *dsrAB* sequences generated in this study have been deposited in GenBank under accession numbers KP118849-KP118879.

Clone sequences were masked to contain only unambiguously aligned nucleotide positions (268 nucleotides; 329 of 336 sequences), and subsequently clustered at a 97% sequence similarity cut-off value using the average neighbor clustering method as implemented by the mothur software package (Schloss et al., 2009, Figure S2). Gene richness, evenness, and diversity were assessed by the Chao1 richness estimator (S_chao1_) (Chao, 1984), Simpson evenness index (E_simpson_) (Simpson, 1949), and the non-parametric Shannon diversity index (H_shannon_) (Shannon, 1948), respectively, as implemented in mothur (Schloss et al., 2009) (Table S3).

### Enumeration of dsrAB gene abundance via quantitative PCR

The abundance of sulfate-reducing microbes in basaltic fluid samples was investigated using quantitative PCR (qPCR) of *dsr*. Gene copy quantification was performed with the Eppendorf Mastercycler® ep realplex real-time PCR System (software version 2.2) (Eppendorf, Hauppauge, NY, USA). qPCR reaction mixtures (20 μl) were prepared in triplicate using the KAPA SYBR® FAST Master Mix Universal qPCR kit with low ROX (Kapa Biosystems, Woburn, MA, USA) using previously developed *dsrA* gene specific primers DSR1F+ (5′-ACSCACTGGAAGCACGGCGG-3′) and DSR-R (5′-GTGGMRCCRTGCAKRTTGG-3′) (Kondo et al., 2004) (Table S1), and 1 μl of template environmental DNA. The thermal cycling conditions consisted of an initial denaturation at 94°C for 10 min followed by 35 cycles of 94°C denaturation for 1 min, 65°C annealing for 1 min, 72°C elongation for 30 s, and fluorescence read after 10 s at 80°C. Following amplification, purity of the product was assessed by viewing temperature dissociation curves determined across a range of temperature values (55–95°C).

Quantification was based on a standard curve generated from genes amplified from plasmids containing full-length *dsrAB* amplicons, which were serially-diluted from 10^11^ to 10^1^ copies μl^−1^. Plasmids were generated from picked colonies produced during PCR of full-length *dsrAB* genes, which were grown overnight in LB media and extracted using the QIAprep Spin Miniprep Kit (Qiagen). Following the extraction, qPCR primers were used to amplify the target gene sequence using a thermocycling PCR protocol identical to as described in the Dissimilatory (Bi)Sulfite Reductase (dsrAB) Gene Cloning and Sequencing Section. PCR products were quantified using the Quant-iT™ dsDNA Assay High Sensitivity Kit (Q33120, Life Technologies, Carlsbad, CA, USA).

### 16S rRNA gene amplification, sequencing, and analysis

In order to further characterize the microbial communities within fluid samples from boreholes U1301A and 1025C, we used an approach that involves amplifying a region of the 16S rRNA gene from environmental DNA using primers that are specific to bacteria and archaea and sequencing on the Illumina MiSeq platform (Caporaso et al., 2011). The V4 region of the 16S rRNA gene was amplified with primers 515f and 806r that include the Illumina flowcell adapter sequences. The reverse (806r) primer also contains an additional 12-bp barcode, which is used to assign individual sequences to samples. Triplicate PCR reactions were pooled at equimolar concentrations and PCR cleanup was performed on the final pooled product using the UltraClean PCR clean up kit (MoBio Laboratories, Carlsbad, CA, USA). Sequencing was performed on an Illumina MiSeq sequencer (Illumina, Inc., San Diego, CA, USA) at the University of Colorado BioFrontiers Institute.

The resulting 16S rRNA gene sequences were processed using the SILVAngs analysis pipeline (Quast et al., 2013), which performs processing of the data via five basic modules: alignment against the SILVA SSU rRNA SEED using the SILVA Incremental Aligner (SINA) (Pruesse et al., 2012), quality control, dereplication, and clustering using CD-HIT (Li and Godzik, 2006) at a 98% sequence similarity level, and classification using BLAST search against the non-redundant version of the SILVA SSU Ref dataset release 111 using blastn with standard settings (Camacho et al., 2009). Following identification of the microbial lineages, greater than 40,000 gene sequences related to know SRM were detected. Sample metadata and the SSU rRNA sequence files used in this study have been submitted to the NCBI BioSample and Sequence Read Archive databases and can be accessed using the BioProject identifier PRJNA266850.

## Results and discussion

### Sampling and hydrogeochemical setting

**Figure 1:**
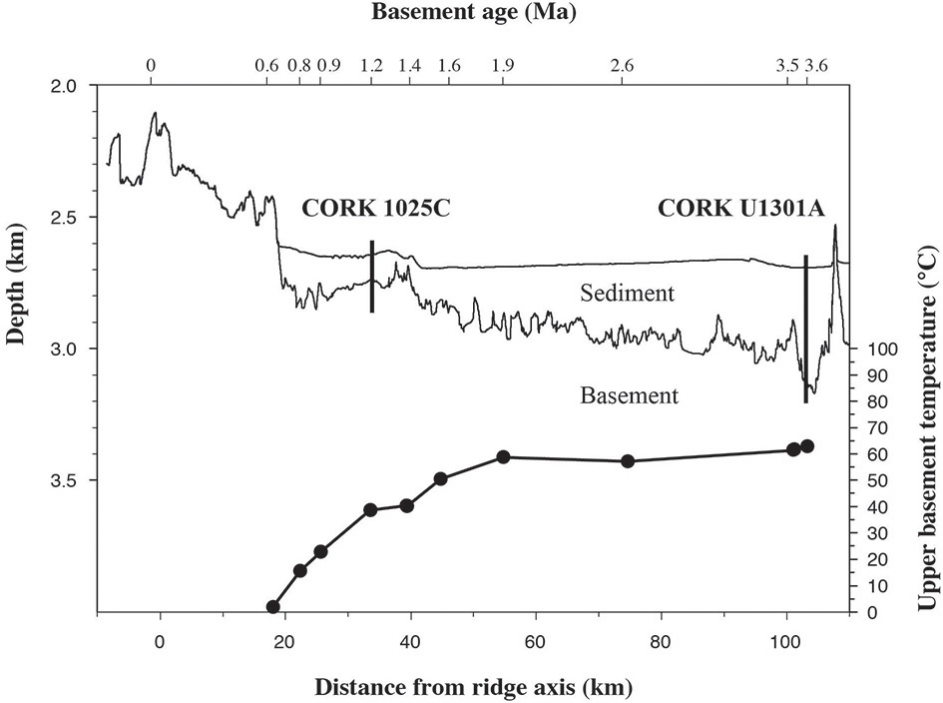
**Interpreted composite cross section of the JFR from active spreading center to the west showing sediment thickness, basement topography and age, and temperature**. Data from selected results from ODP leg 168 and related experiments (Fisher et al., 2000). Locations of CORKs 1025C and U1301A are indicated.

High integrity basaltic fluid samples were collected from CORKs installed at increasing distance from the spreading ridge axis fitted within boreholes 1025C and U1301A (Figure 1). Borehole 1025C (47°88.7′N, 128°64.8′W) is situated 14 km from the transition between sediment-free and sediment-buried basement penetrating 101 m of sediment and the upper 46 m of basement (in crust, 1.2 Myr old). Also fitted with a CORK, borehole U1301A (47°45.2′N, 127°45.8′W) is situated 68 km to the east of borehole 1025C and drilled 101 km east of the JFR spreading center, cased through 262 m of sediment and penetrates the upper 108 m of basement (in crust, 3.5 Myr old). Relative to deep-sea bottom seawater, the fluids collected from borehole 1025C are depleted in dissolved organic carbon (DOC), oxygen, nitrate, and sulfate and enriched in reduced species such as hydrogen sulfide and methane (Table 1; Lin et al., 2012; Jungbluth et al., 2014). In comparison to 1025C fluids, the fluids collected from borehole U1301A are characterize by a stronger chemical alteration (Table 1; Lin et al., 2012; Jungbluth et al., 2014), demonstrating a gradual decline in redox potential of the oxidants, from oxygen to sulfate, which is accompanied by a decrease of the organic material concentration in fluids. These observations are consistent with the thermodynamic sequential model for electron acceptors (Jørgensen, 2006) and indicate a geochemical sequence associated to a decrease in the free energy available to support microbial life in deep subsurface fluids.

### Rates of microbial sulfate reduction

**Figure 2:**
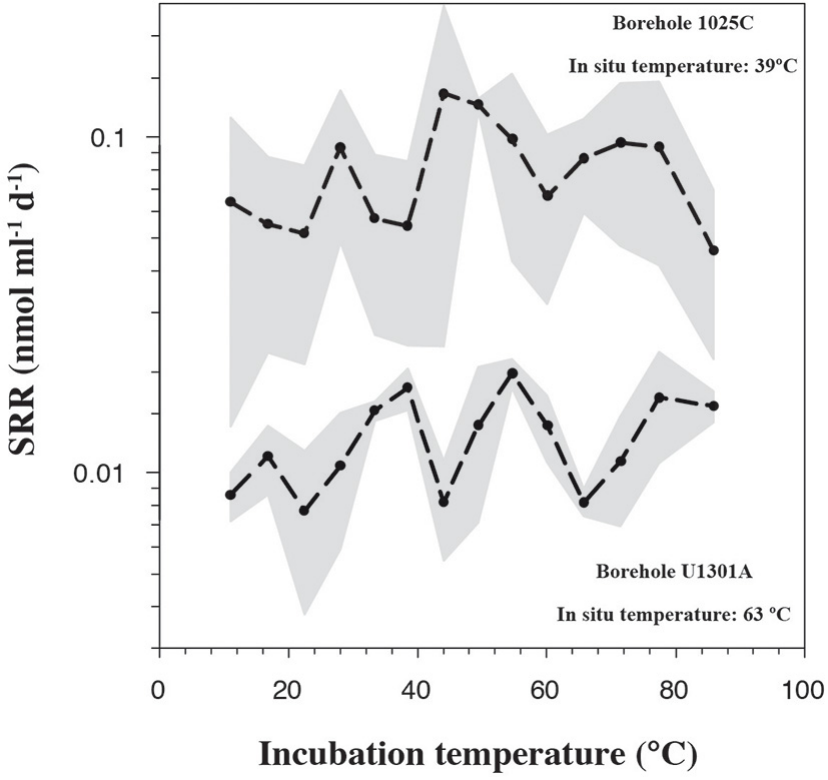
**Sulfate reduction rates, determined from temperature-gradient experiments after 3 and 5 days of incubation of unamended crustal fluids collected from boreholes 1025C and U1301A, respectively**. Full circles represent the average of two experimental replicates. The range between maximum and minimum measurements is shaded in gray.

**Figure 3:**
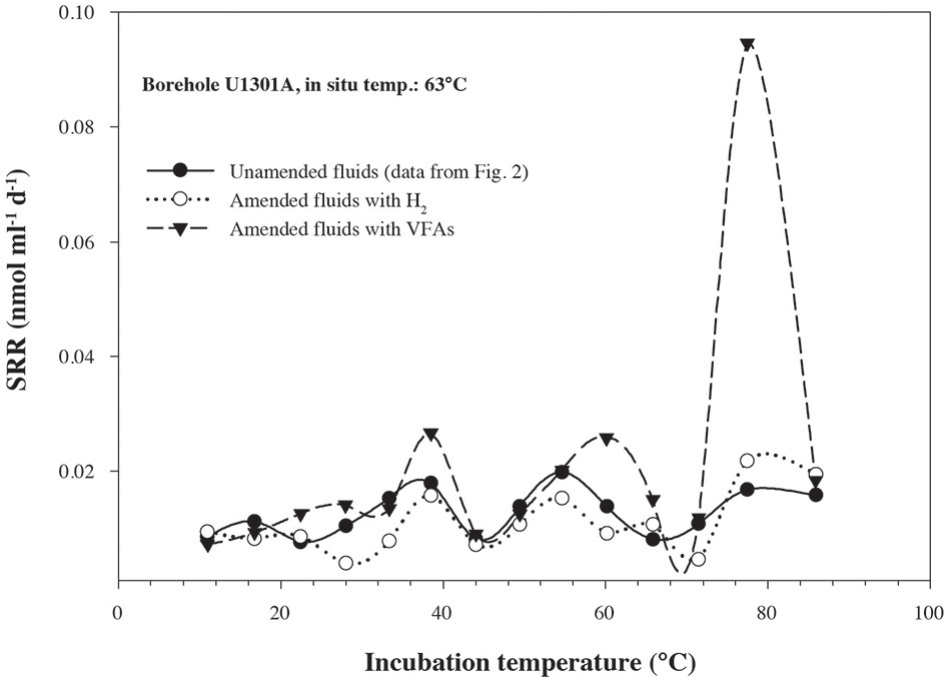
**Sulfate reduction rates, determined from temperature-gradient experiments after 3–5 days of incubation of; unamended crustal fluids collected from borehole U1301A, amended U1301A fluids with a mix of short-chain volatile fatty acids (VFAs) at 1 mM concentration, amended U1301A fluids with hydrogen at near saturation**. Data points represent the average of two experimental replicates.

**Figure 4:**
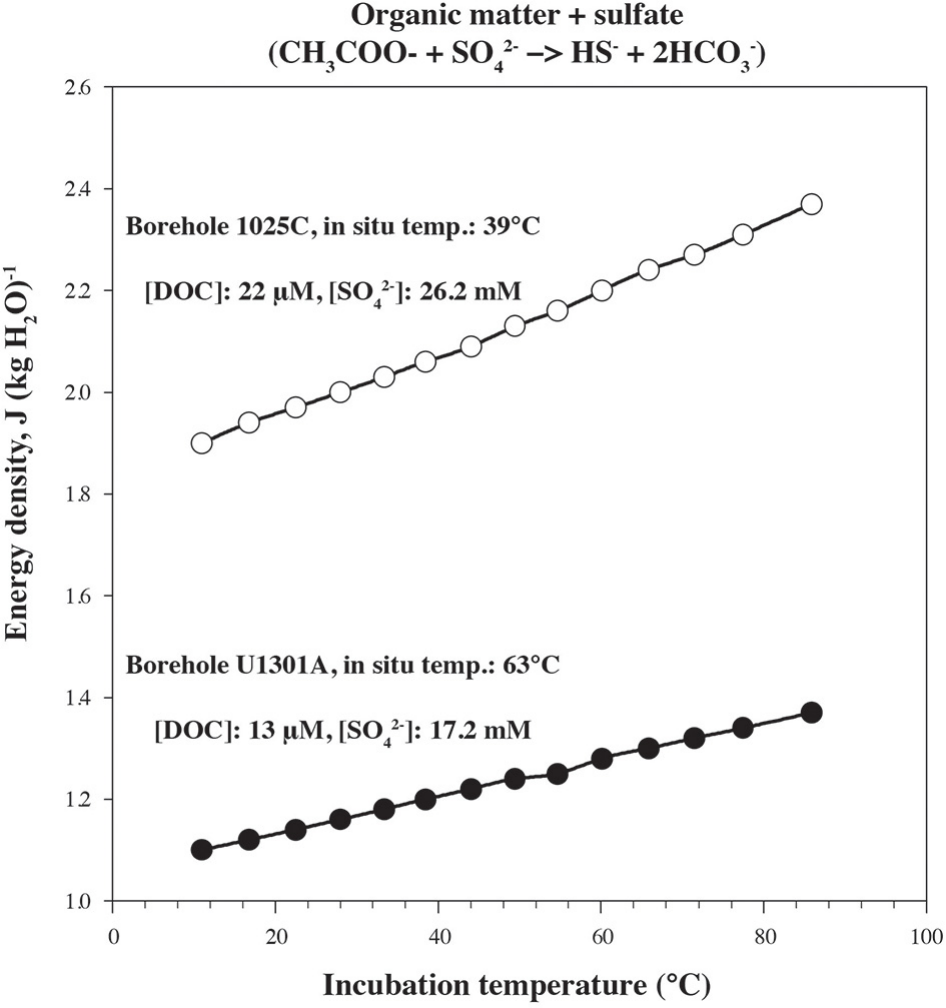
**Gibbs energy of reaction for organic matter being completely oxidized by sulfate in borehole 1025C (open circles) and U1301A (full circles) fluids as a function of temperature normalized per kg of H_2_O**.

In order to quantify microbial activity in basaltic fluids,^35^SO^2−^_4_ radiotracer incubation experiments were conducted in duplicates at 14 temperature increments between +10°C to +86°C using a thermal gradient block. Sulfate reduction was detected at all temperatures investigated, providing the first account of potential microbial activity in deep sub-seafloor basaltic fluids (Figure 2). Sulfate reduction rates (SRR) in unamended incubations at *in situ* temperatures were higher in lower temperature fluids from borehole 1025C (0.05 nmol ml^−1^ d^−1^ at 39°C) compared to in higher temperature fluids from borehole U1301A (0.01 nmol ml^−1^ d^−1^ at 63°C). The younger upper oceanic crust of JFR (borehole 1025C) experiences a more vigorous recharge of deep-sea bottom seawater through unsedimented areas than the older crust (borehole U1301A), which is covered by a thicker sediment layer (Johnson et al., 2006). Active recharge of the crustal aquifer with fresh bottom seawater may replenish the microbial communities near borehole 1025C with electron donors more so than those in older crust near borehole U1301A. This potential decreasing availability of electron donors across the JFR flank was further investigated by amending U1301A fluids with H_2_ or a mixture of organic acids (acetate, butyrate, propionate, and lactate). SRR in organic acid-amended samples were consistently higher than in H_2_-amended and unamended samples, with the largest difference—almost an order of magnitude—observed at 77°C (Figure 3). This suggests that predominantly organotrophic sulfate-reducing communities in crustal fluids are inherently electron donor limited. At the *in situ* concentrations of DOC and sulfate (Figure 4; Table 1), calculated Gibbs energies are nearly twice as exergonic in younger crust (1025C) than in the more highly reacted, nutrient-depleted fluids (U1301A). This decreasing energy density explains the difference in measured potential SRR between the two samples.

### Temperature characterization of sulfate-reducing microorganisms

Apparent temperature optima (T_opt_), here defined as the temperature with the maximum ^35^S-SRR, were detected at around 28–38, 44–55, and 71–77°C (Figure 2). Note, that SRM can have their highest SRR 5–10°C above their T_opt_ for growth (Knoblauch and Jørgensen, 1999). Observed T_opt_ are therefore interpreted as metabolic activity of coexisting meso- and thermophilic groups of microbial sulfate reducers (Elsgaard et al., 1994) and implies the survival of microorganisms at *in situ* fluid temperatures outside their optimum range. While the observation of SRR at high incubation temperatures in temperate fluids from 1025C could reflect the induced germination of thermophilic endospores (Müller et al., 2014) that are otherwise inactive at their respective *in situ* temperatures of 39°C, mesophilic SRR in 63°C fluids from U1301A may partly be explained by cold bottom seawater intrusions (Jungbluth et al., 2014). Mixing of populations with different thermal tolerances may be facilitated by rapid advective fluid flow (80–120 m/h) in the uppermost basaltic crust at the JFR flank (Fisher et al., 1997). Nevertheless, SRR in fluids from 1025C are highest at mesophilic temperatures while those from U1301A are highest at thermophilic temperatures. For example, in unamended 1025C fluids, the maximum SRR (0.14 nmol ml^−1^ d^−1^) was at 44°C (Figure 2); this is only a few degrees higher than the *in situ* borehole temperature of 39°C. In contrast, SRR optima in unamended U1301A fluid incubations were higher by ~6–10°C, and peaked at ~77°C (0.02 nmol ml^−1^ d^−1^, Figure 2).

### Functional diversity

**Figure 5:**
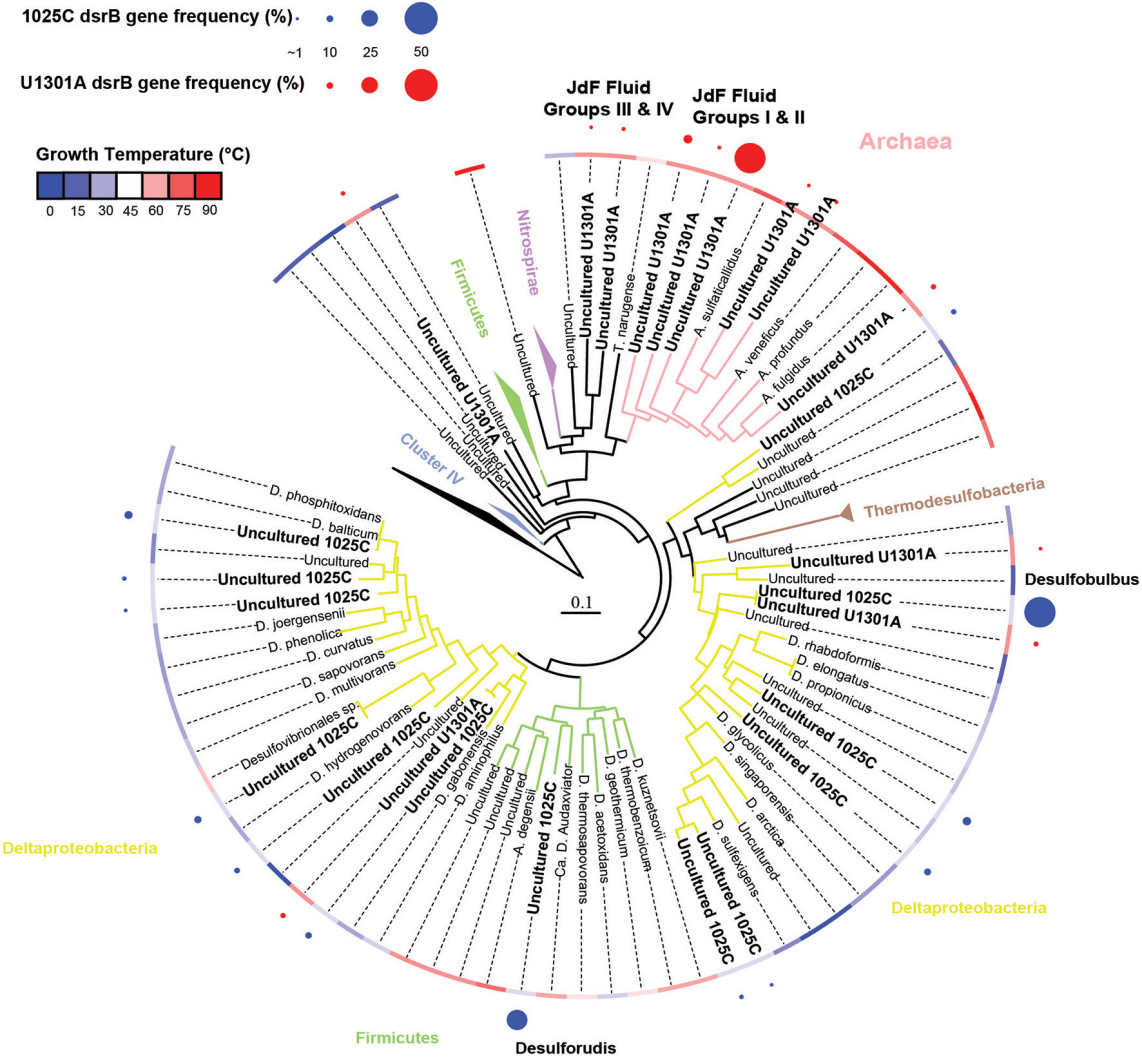
**Phylogenetic relationships of *dsrB* sequences from borehole 1025C and U1301A fluids**. Gene clones identified in this study are highlighted in bold; the relative abundance of identical clones recovered from the same sample is indicated by circle size. Tree branches are colored according to the lineage names that are listed. The blue-red color temperature gradient represents the optimum growth temperature of cultivated strains, or in the case of cloned sequences, the average temperature of the sampled environment. Reverse dissimilatory sulfite reductase genes were used as an outgroup.

**Figure 6:**
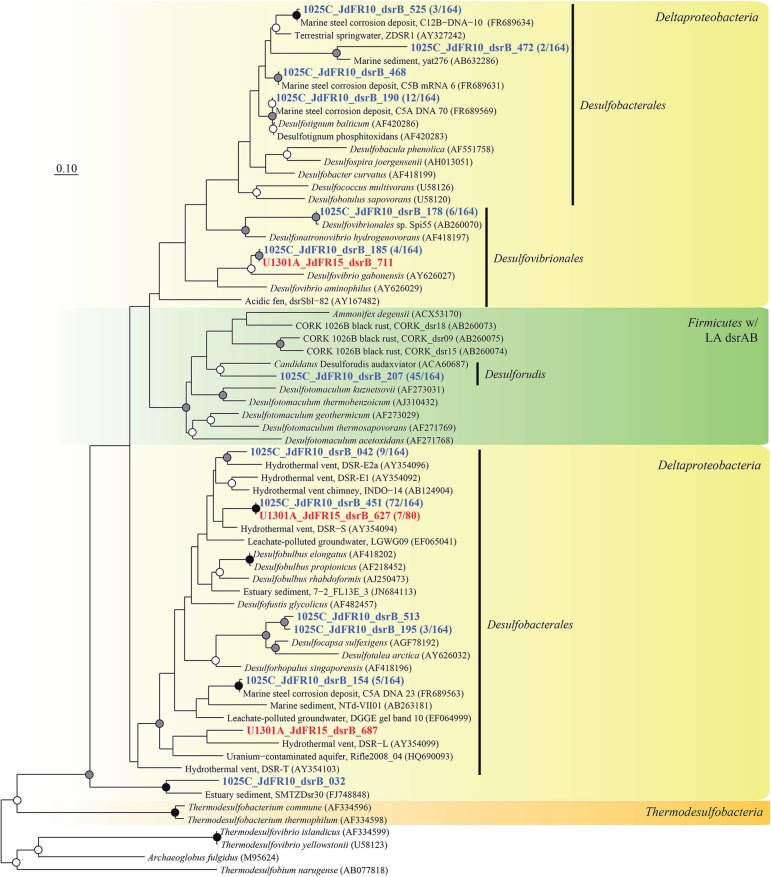
**Phylogenetic relationships of borehole fluid *dsrB* fragments from the *Deltaproteobacteria* class and *Firmicutes* phylum containing laterally acquired *dsrAB***. Gene clones recovered in this study are highlighted in bold font; the fractional abundance of identical clones recovered from the sample is listed in parentheses. Black (100%), gray (>80%), and white (>50%) circles indicate nodes with bootstrap support, from 1000 replicates. The scale bar corresponds to 0.1 substitutions per nucleotide position.

**Figure 7:**
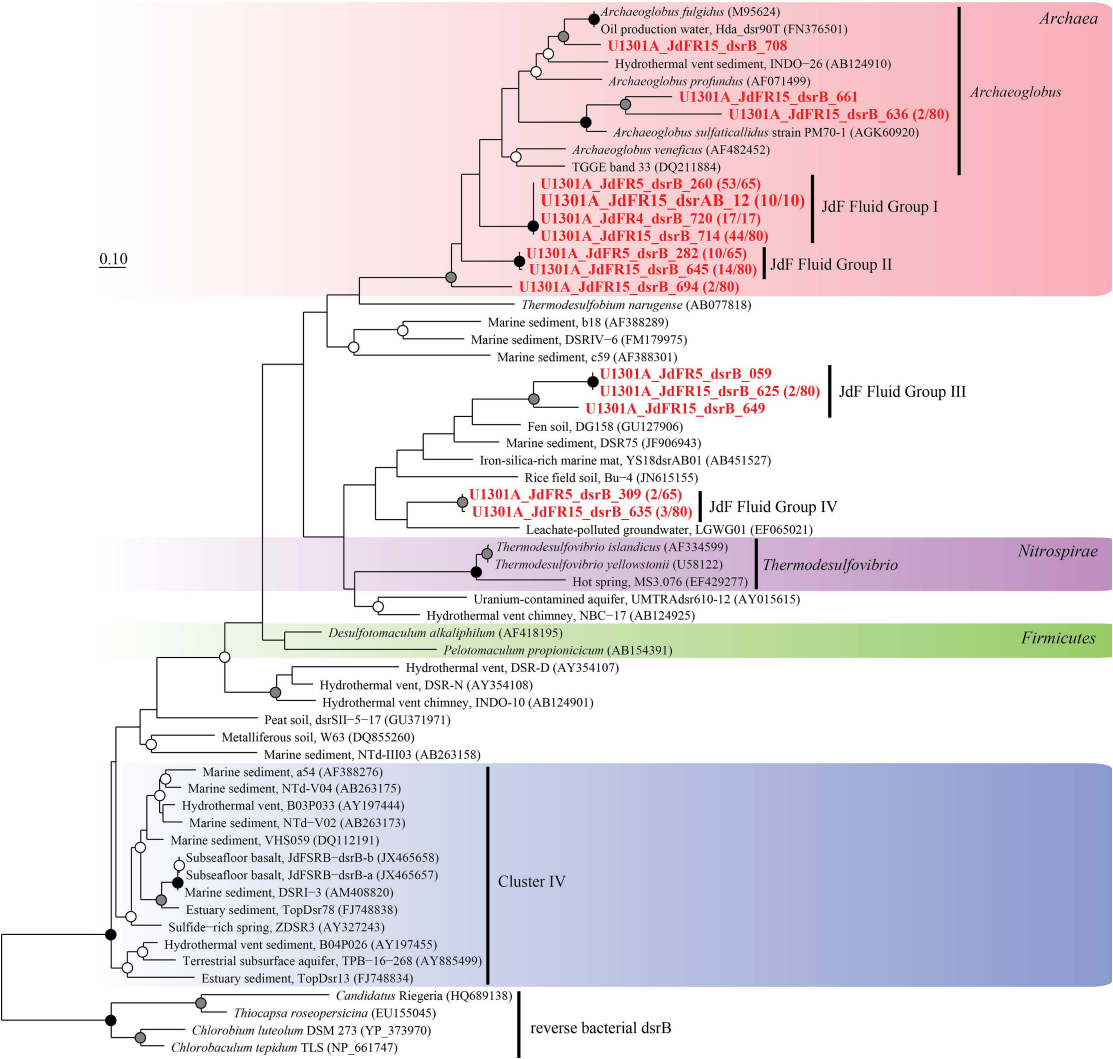
**Phylogenetic relationships of borehole fluid *dsrB* fragments from the domain *Archaea* and uncultivated lineages**. Gene clones recovered in this study are highlighted in bold font; the fractional abundance of identical clones recovered from the sample is listed in parentheses. Black (100%), gray (>80%), and white (>50%) circles indicate nodes with bootstrap support, from 1000 replicates. The scale bar corresponds to 0.1 substitutions per nucleotide position.

**Figure 8:**
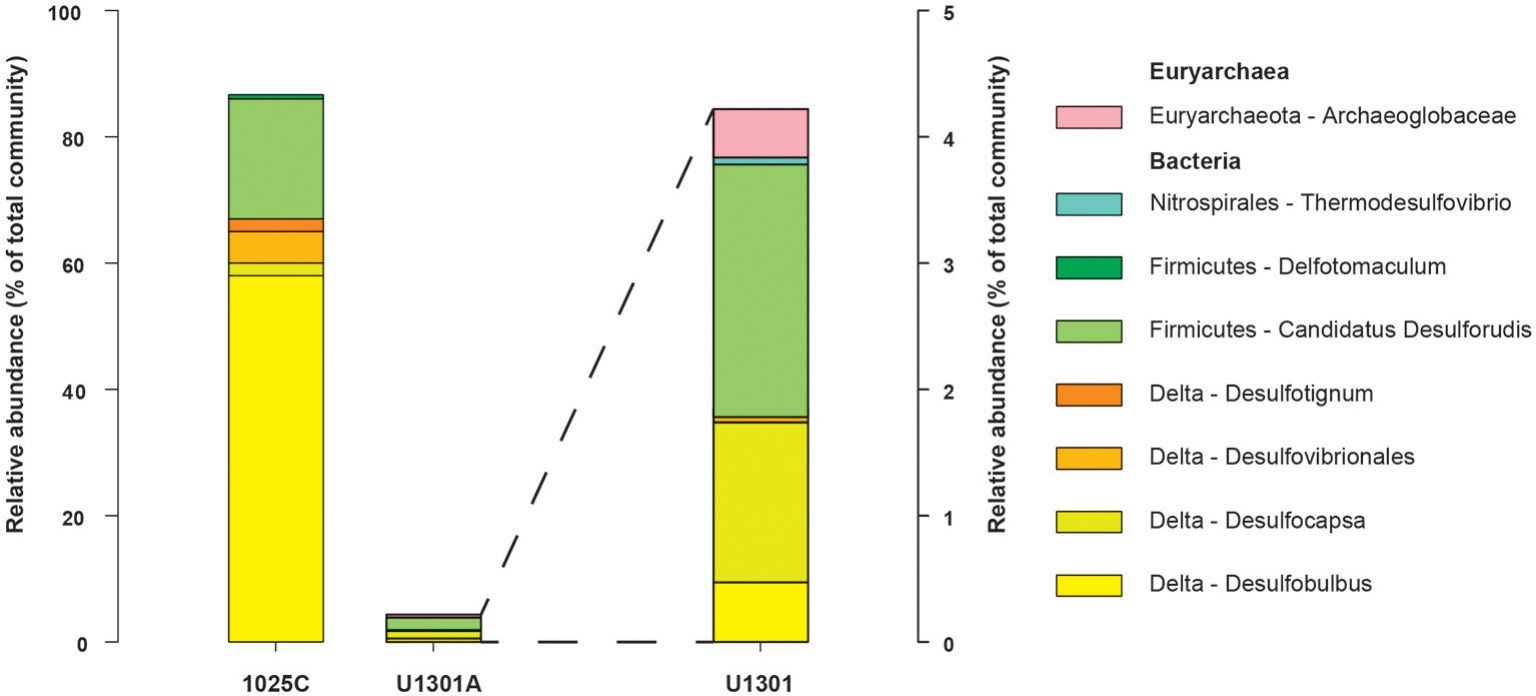
**SSU rRNA gene relative abundance from putative SRM recovered from borehole 1025C and U1301A fluids as inferred from close phylogenetic relationships to known sulfate-reducers**.

The physiological temperature characterization of sulfate-reducing microbial communities in the fluids is supported by the sequence diversity of the functional marker gene *dsrB*. The *dsrB* found in borehole 1025C (*in situ* temperature of ~39°C) are more closely related to known mesophiles and moderate environmental temperatures, whereas *dsrB* from U1301A (63°C) are indicative of thermophiles and warmer environments (Figures 5–7). Only two of 31 classified operational taxonomic units within the bacterial orders *Desulfovibrionales* and *Desulfobacterales* were shared among 1025C and U1301A fluids. Clone libraries of *dsrB* from crustal fluids of 1025C revealed SRM distributed within the class *Deltaproteobacteria* and phylum *Firmicutes*, predominantly belonging to the order *Desulfobacterales* (55%) and *Candidatus* Desulforudis (27%), respectively (Figures 5, 6). *Candidatus* Desulforudis is an uncultured gram-positive bacterium observed in mesophilic (here and Jungbluth et al., 2014) and thermophilic (Jungbluth et al., 2013) crustal fluid habitats of the JFR, and prevalent in hot terrestrial subsurface fluids (Davidson et al., 2011); genomic analysis revealed it not only to be well suited for the isolating conditions within the Earth's crust but also to have the ability to form endospores (Chivian et al., 2008). The stimulation of endospore germination observed after the incubation of temperate fluids from 1025C (see discussion above) may constitute the first evidence linking sulfate reduction activity with the marine Desulforudis lineage. In contrast to 1025C, the majority of the U1301A *dsrB* sequences (93%) had no close cultivated relatives, and instead clustered into four monophyletic lineages (named JFR Fluid Group I–IV; Figures 5, 7). JFR Fluid Groups I and II accounted for 84% of the U1301A *dsrB* sequences and appear to be of archaeal origin (Figure 5 and Figure S1), but share only 70% nucleotide sequence similarity with the closest related hyperthermophilic *Archaeoglobus* isolate. A higher frequency of *Archaea* is commonly observed in other energy-starved subsurface environments (Biddle et al., 2006; Lipp et al., 2008; Roussel et al., 2008; Schippers et al., 2010). Under the persistent extreme conditions found in basaltic fluids of borehole U1301A (e.g., high temperature and low-energy), the *Archaea* harboring these divergent *dsrB* variants likely catalyze functions that in fluids from 1025C are largely ascribed to bacteria. The proportion of small subunit (SSU) ribosomal RNA gene sequences related to known sulfate reducers differed between the two boreholes, with 87% in 1025C and 4% in U1301A (Figure 8). This supports the emergent presence of novel archaeal lineages in fluids from U1301A not amplified by currently available 16S rRNA primers. Notably, many lineages were detected that are unrelated to those attached to basaltic surfaces (Lever et al., 2013; Cluster IV, Figure 5), further suggesting that novel sulfate-reducing lineages predominate highly altered crustal fluids.

### Cell-specific respiration rates of SRM

Assuming that each sulfate-reducing cell contains one copy of *dsrAB* (Klein et al., 2001; Kondo et al., 2004) leads to maximum estimates of 1.6 × 10^4^ and 2.5 × 10^2^ sulfate-reducing microbial cells ml^−1^ in 39°C and 63°C fluids, respectively. Based on our measured potential SRR in unamended experiments under *in situ* temperatures, cell-specific SRR in crustal fluids range from 3.3 to 56 fmol cell^−1^ day^−1^. Comparatively, higher apparent cell-specific SRR in 63°C fluids from U1301A indicate that, if microbial communities in crustal fluids are indeed living at their basal power requirement (sensu; Hoehler and Jørgensen, 2013), the costs of maintenance may increase with temperature. These cell-specific SRR are however higher than those measured in depositional marine settings (D'Hondt et al., 2002; Hoehler and Jørgensen, 2013) and conspicuously similar to laboratory batch cultures (Knoblauch et al., 1999), suggesting that laboratory incubations may have stimulated the rates of sulfate reduction. Incubation experiments at atmospheric pressure can result in higher rates of microbial metabolism (Whelan et al., 1986), however current views suggest that metabolic measurements made using decompressed samples tend to grossly underestimate *in situ* activity (Picard and Daniel, 2013; Tamburini et al., 2013). Entrainment of bottom seawater into gas-tight samplers could hypothetically also enhance the laboratory rates of sulfate reduction in basaltic fluids with the replenishment of substrates, but the low DOC concentrations in basement fluids demonstrate good control over sampling (Lin et al., 2012). The reasons for a potential overestimation of gross *in situ* rates with the amendment of ^35^SO^2−^_4_ are uncertain and yet, radiotracer techniques appear to overestimate the metabolic potential of microorganisms in deep aquifer systems (Chapelle and Lovley, 1990). It is therefore difficult to extrapolate the cell-specific metabolic activity of SRM to *in situ* conditions. Nonetheless, our measurements demonstrate that, as seen in other deeply buried subsurface sedimentary microbes (Morono et al., 2011), SRM in basaltic fluids maintain high potential for metabolic activity under different environmental conditions.

### Ecological implications

The upper oceanic crust is arguably the most favorable of deep-subsurface environments due to the vigorous production hydrothermal fluids and sporadic recharge of bottom seawater through unsedimented areas (Johnson et al., 2006). In comparison, our SRR are an order of magnitude higher than the potential SRR of ~0.002 nmol ml^−1^ d^−1^ measured for sediments at the sediment-basement interface (~262 mbsf) nearby U1301A (Engelen et al., 2008). However, the question remains, do the high rates of sulfate reduction measured in this study correspond to *in situ* SRR? We have demonstrated that organic material is mineralized anaerobically by SRM in basaltic fluids as indicated by the stimulation of sulfate reduction after the amendment with short-chain organic acids. Such small organic compounds accessible to sulfate reducers for direct mineralization may be made available from DOC by hydrolytic and fermentative members of the phylum *Bacteroidetes* (~11% and 4.7% of the total 16S rRNA gene sequences in fluids from 1025C and U1301A, respectively), and by a potential temperature activation of organic matter (Wellsbury et al., 1997; Parkes et al., 2007). DOC represents most organic matter carried by crustal fluids and its chemical and isotopic composition suggests its quantitative removal during transit in the JFR flank (McCarthy et al., 2011). It can therefore be calculated that, assuming a stoichiometry of two moles of organic carbon per mole of SO^2−^_4_ (D'Hondt et al., 2002; Bowles et al., 2014), potential SRR measured in the basement fluids of 1025C and U1301A yield mineralization rates of organic carbon by sulfate of 0.1 to 0.02 nmol ml^−1^ d^−1^, respectively. These rates are over four to five orders of magnitude greater than the recharge-based estimates of net DOC removal rates in basement fluid samples from 1025C and U1301A, 6.0 × 10^−6^ and 6.5 × 10^−6^ nmol ml^−1^ d^−1^, respectively (Lin et al., 2012). However, above calculations of net DOC removal rates only consider the amount of deep ocean DOC entering the system at basement recharge zones. There are additional sources of DOC that could alleviate the restriction of reduced carbon for SRM and support high SRR in basaltic fluids, e.g., autochthonous DOC synthesis from inorganic carbon by chemosynthetic crustal microbial communities (McCarthy et al., 2011), and diffusion of DOC from sediments to basaltic fluids (Lilley et al., 1993; Party and Johnson, 2003). The present study indicates that sulfate reduction is sufficient to explain much of the net removal of residual deep ocean DOC in fluids of the JFR. Because fluid flow regimes in the JFR flank are representative of conditions that are common in all ocean basins (Party, 1997), it follows that microbial sulfate reduction in basaltic fluids plays a qualitatively and quantitatively significant role in the global biogeochemical carbon cycling between the subsurface and the overlying ocean.

### Conflict of interest statement

The authors declare that the research was conducted in the absence of any commercial or financial relationships that could be construed as a potential conflict of interest.
