# Supplementary material for: Activity and phylogenetic diversity of sulfate-reducing microorganisms in low-temperature subsurface fluids within the upper oceanic crust
Source: Front Microbiol. 2015 Jan 14;5:748. doi: 10.3389/fmicb.2014.00748 (PMC4295021; doi:10.3389/fmicb.2014.00748)
Supplement: Supplementary file 4 [file Image1.PDF]

Supplementary Material

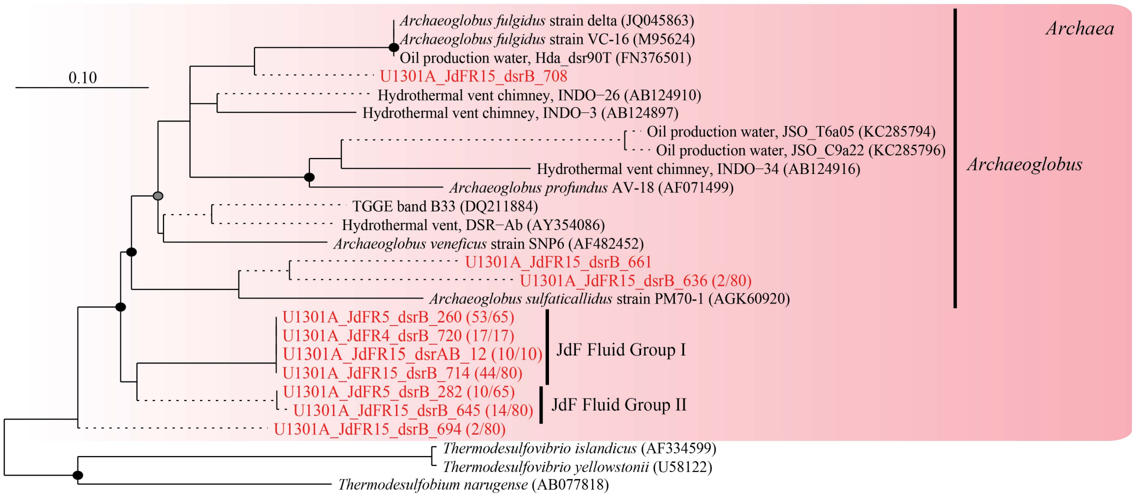

Figure S1. Phylogenetic relationships of borehole fluid *dsrB* and gene fragments from the domain *Archaea*. The backbone of the phylogenetic tree was generated using full-length *dsrB* sequences. Relevant gene clones of short length and gene clones fragments described in this study were added after tree construction and bootstrapping, and are indicated by dashed lines. Gene clones recovered in this study are highlighted in bold font; the fractional abundance of identical clones recovered from the sample is listed in parentheses. Black (100%), grey (>80%), and white (>50%) circles indicate nodes with bootstrap support, from 1000 replicates. The scale bar corresponds to 0.1 substitutions per nucleotide position.

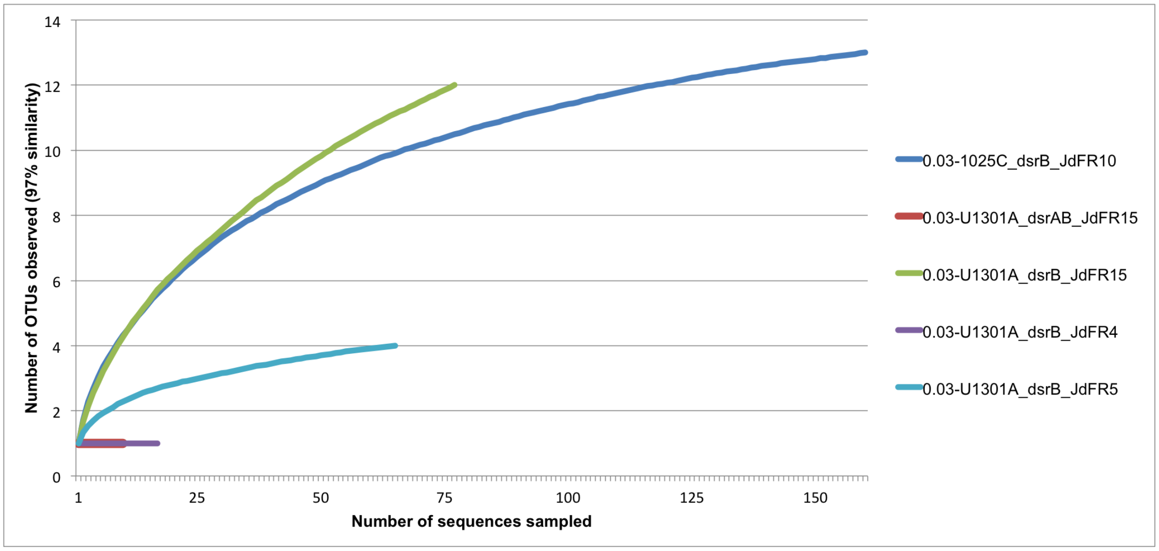

Figure S2. Rarefaction curves displaying the number of operational taxonomic units (OTUs) observed given the sequencing effort for each sample, clustered at 97% sequence similarity cut-off values.
